# Simultaneous infection of *Schistosoma mansoni *and *S. rodhaini *in *Biomphalaria glabrata*: impact on chronobiology and cercarial behaviour

**DOI:** 10.1186/1756-3305-1-43

**Published:** 2008-12-02

**Authors:** Alice Norton, David Rollinson, Louisa Richards, Joanne Webster

**Affiliations:** 1 Department of Infectious Disease Epidemiology, Imperial College Faculty of Medicine, London, W2 1PG, UK; 2 Wolfson Wellcome Biomedical Laboratories, Department of Zoology, The Natural History Museum, London, SW7 5BD, UK

## Abstract

**Background:**

The chances of a schistosome cercaria encountering a suitable definitive host may be enhanced by emergence from the molluscan intermediate host with maximal glycogen stores and by an appropriate chronobiological rhythm. This study aimed to identify and characterize the effects of potential competitive interactions in the snail host *Biomphalaria glabrata*, between the closely-related *Schistosoma mansoni *and *S. rodhaini*, on phenotypic behavioural traits. It was predicted that inter-specific competition would affect chronobiological emergence rhythms and reduce the activity of schistosome swimming behavioural traits. *Biomphalaria glabrata *snails (120) were exposed to either *S. mansoni *or *S. rodhaini *single infections, or a mixed infection of both species simultaneously and the resulting cercarial phenotypic traits were characterised. Cercariae were identified from co-exposed snails by amplification and sequencing of the mitochondrial cytochrome oxidase subunit 1 (CO1).

**Results:**

*S. mansoni *and *S. rodhaini *largely maintained their distinct chronobiological rhythms after mixed exposures and infections. However, inter-specific competition appeared to result in a restriction of the shedding pattern of *S. rodhaini *and slight shift in the shedding pattern of *S. mansoni*. Inter-specific competition also significantly lowered hourly cercarial production for both parasite species in comparison to single exposures and infections and reduced cercarial swimming activity.

**Conclusion:**

Inter-specific competition was shown to influence cercarial production, chronobiology and activity and should therefore be investigated further in field situations to determine the effects of these changes on parasite fitness (incorporating both host finding and infectivity) where these two species overlap. Importantly this competition did not result in a large change in chronobiological emergence of cercariae for either species indicating that it would not have a large influence on the species of hosts available for infection at time of emergence. This study has furthermore demonstrated the potential for phenotypic measures to provide markers for species-specific identification even in conditions of co-infection.

## Background

Transmission between hosts is a vital part of the life-cycle for any parasitic organism. For many parasitic platyhelminths such transmission is achieved by free-living larval stages, which have limited energy reserves and short life-spans. Strong selection pressures are therefore expected to influence the phenotypic behaviour of such larvae in order to improve their transmission success.

Schistosomes of the genus *Schistosoma *are the causative agent of schistosomiasis, a parasitic disease second only to malaria in terms of its socio-economic and public health importance [1]. They are platyhelminth macroparasites with an indirect life-cycle involving a mammalian and molluscan host. Transmission between the two occurs via free swimming larval stages: cercariae (infective to the mammalian definitive host) and miracidia (infective to the molluscan intermediate host). Schistosome cercariae have a well developed tail with longitudinal striated muscles extending underneath the tegument with which they swim to find their definitive hosts. Glycogen stores within this tail provide a non-renewable energy source which is depleted during swimming [2]. Cercariae are therefore adapted to maximise their chance of encounter with a host by, for instance, emerging in the correct 'host-time' as well as 'host-space' and such adaptations may be specific to their definitive host species [2-4]. Cercarial swimming behaviours have been shown to be influenced by several stimuli such as light levels and host chemical signals [5,6] Biotic pressures, for example competition from other species of parasites for shared resources within the same host, are also be predicted to reduce the glycogen reserves [7] and hence subsequent activity and infectivity of those cercariae shed [7,8].

*S. mansoni *and *S. rodhaini *are closely related species within the lateral-spined *S. mansoni *group [9]. Their larval stages are morphologically similar and therefore difficult to distinguish visually. Furthermore, their host ranges may overlap in both the intermediate host (freshwater snails of the genus *Biomphalaria*) and the definitive host. *S. mansoni *can infect both primates and rodents [10] and *S. rodhaini *can infect rodents and canines [11,12] and they are known to co-occur and co-infect in parts of Africa [13-16]. Their main definitive hosts, however, differ being humans for *S. mansoni *and rodents for *S. rodhaini*. The cercarial behavioural repertoire of these two species may therefore be expected to differ to enhance transmission to these contrasting definitive hosts. Indeed, *S. mansoni *cercariae have been demonstrated by several authors to exhibit a diurnal shedding pattern and *S. rodhaini *cercariae a nocturnal shedding pattern therefore linking the timing of cercarial emergence in nature to coincide with the highest activity of their main definitive host species in the water [12,13,17]. Interestingly, a recently published field study reported, that *S. rodhaini *from Kenya can exhibit a bimodal cercarial emergence profile, with peaks between both 0500 and 0800 and also between 1900 and 2200 [16].

Under conditions where these two parasite species interact and compete, such interactions are predicted to influence their behaviour. The chronobiology of laboratory-bred hybrids of these two species has previously been investigated [17] as well as intra-specific hybrids of two strains of *S. mansoni *with different shedding peaks from Guadeloupe [18] and these have both shown chronobiology to be genetically determined. Furthermore, the chronobiology of *S. mansoni *and *Ribeiroia marini *cercariae emerging from co-infected *Biomphalaria glabrata *[19] and *S. haematobium *and *S. bovis *cercariae emerging from co-infected *Bulinus truncatus *[20] have been investigated and small shifts in the parasite's chronobiology have been found. The aforementioned recent field study, which investigated the chronobiology of *S. mansoni *and *S. rodhaini *emerging from one co-infected snail, reported that co-infection did not affect either species' cercarial emergence patterns when measured phenotypically [16]. However, data from adult worms obtained by the infection with these cercariae of mice indicated that there may be an effect of co-infection on *S. rodhaini's *emergence [16]. No previous study has, however, been able to identify emerging cercariae to species using molecular techniques, meaning that for time periods where cercarial shedding of the two species overlap effects of competition may not have been accurately identified.

The main aim of this study was to use new molecular techniques [21] to accurately characterize the impact of any inter-specific competition between these species on cercarial emergence and to observe the impact on swimming activity. The elucidation of such behavioural repertoires is important for understanding the mechanisms of host location for parasites in general as phenotypic plasticity may be vital in determining a parasite's ability to infect the next host stage.

## Methods

### Host-parasite maintenance

Parasites used in these experiments were well established strains of *S. mansoni *and *S. rodhaini *which had been passaged routinely in the laboratory through *B. glabrata *(a species well adapted for laboratory culture and closely related to the natural *Biomphalaria spp*. hosts of the parasite species, the latter of which are not well adapted for laboratory culture and provide prohibitively small sample sizes for experimental models). All snails were maintained in the laboratory at 23–25°C and subjected to a 12 L (07:00–19:00): 12 D (19:00–07:00) light regime (full ultra-violet spectrum, Sun-glo natural sunlight lamps). Snails were housed individually in plastic pots (10 cm × 8 cm × 5 cm) in 100 ml of Caledonian spring water (Iceland plc., UK) changed weekly. All snails were fed *ad libitum *on fresh lettuce supplemented with fish food (Tetra Ltd) and chalk.

### Experimental design

Four groups of 30, approximately 10 mm in width and sexually mature (as measured by onset of egg laying), *B. glabrata *were exposed to either 6 *S. mansoni *miracidia, 6 *S. rodhaini *miracidia, 6 miracidia of both species simultaneously or 3 miracidia of both species simultaneously. Individual exposure was performed in 5 ml of spring water for two hours, a period sufficient for maximal miracidial penetration [22].

### Chronobiology

At week seven post exposure of snails to miracidia, the chronobiology of the emerging cercariae was measured. Up to 20 randomly-selected shedding snails from each of the treatment groups were placed in individual vials of 16 ml artificial spring water [23] for 24 hrs in the normal 12 L (07:00–19:00):12 D (19:00–07:00) light regime. The water was replaced every two hours and the number of cercariae shed every two hours estimated from the number of cercariae present in four 0.2 ml samples. Those cercariae shed from snails exposed to both *S. mansoni *and *S. rodhaini *were pelleted by centrifugation and stored in ethanol at – 20°C for each snail at each time point for subsequent molecular sequencing and identification as described below.

### Cercarial swimming behaviour

At weeks five and seven post initial parasite exposure cercariae were shed for two hours in the light (07:00–09:00) and then on a separate day for two hours in the dark (19:00–21:00) to allow for the production of both *S. mansoni *and *S. rodhaini *respectively. At both 09:00 and 21:00 cercariae from individual snails were videoed. This provided six different groups of cercariae from the four snail groups, as those snails which were co-exposed to both *S. mansoni *and *S. rodhaini *were shed at two different time points to allow the shedding of cercariae from both species. A minimum of four randomly selected cercariae (ranging from four to seven) from one snail were placed on a petri dish in 0.15 ml water. They were videotaped (using an Olympus SZ4045 trinocular dissecting microscope attached to a JVC TK-1481 composite colour video camera) for five minutes. Four replicates with new cercariae from each snail were recorded at each time (07:00 and 19:00) on both weeks.

Behavioural categories used for recording were expanded from those described in Norton *et al*., 2007 [5] for *S. mansoni *to include new behaviours observed and further details on behavioural states where the cercariae were attached to the petri dish (see Table 1). Those behavioural categories performed whilst cercariae were unattached were numerically coded and those performed whilst the cercariae were attached were alphabetically coded. Continuous sampling within two second intervals was performed, with viewing order randomised and line identity hidden from the primary observer in order to avoid observer bias. Whilst this method of recording behavioural categories may not accurately reflect the natural environment (where cercariae mainly swim vertically in the water column), previous research has, however, demonstrated such measures to be useful in determining the activity levels of cercariae [7].

**Table 1:** Cercarial behavioural categories.

**Unattached behaviours**	
1	**Straight-line**- fast tail movements resulting in a linear progressive motion. Movement is tail first with the body following precisely in the direction of motion and tail undulating. Random changes in the direction of the motion occur without pause.

2	**Zig- zag line**- fast tail movements resulting in a linear progressive motion with constant changes in direction creating a zigzag pattern. Movement is tail first with the position of the body and tail undulating perpendicularly to the direction of motion. Random changes in the direction of motion occur without pause.

3	**Circular line**- fast tail movements resulting in a progressive motion. Movement is tail first with the body following precisely in the direction of motion. The progressive motion results in movement in a definite circle of measurable diameter with no random direction changes.

4	**Spin**- body and tail spin around the central axis of the cercaria whilst unattached. There is no linear movement

5	**Corkscrew**- body and tail spin around the central axis of the cercaria whilst unattached resulting in a linear progressive motion. The manner of spinning can be described as a 'corkscrew' due to the pattern formed by the forward motion. Random changes in the direction of motion occur without pause.

6	**Drift**- there is no motion of cercaria lasting for more than a brief second, where the cercaria appears to drift unattached.

4/6	**Drifting with intermittent spins**- there is no motion of cercaria lasting for more than a brief second, where the cercaria appears to drift unattached. This is interrupted by spins where the body and tail rotate around the central axis of the cercaria resulting in a tight spin whilst unattached. There is no linear movement

1/6	**Drifting with intermittent forward motion**- there is no motion of cercaria lasting for more than a brief second, where the cercaria appears to drift unattached. This is interrupted by fast tail movements resulting in a linear progressive motion. Movement is tail first with the body following precisely in the direction of motion and tail undulating. Random changes in the direction of the motion occur without pause.

7	**Body first**- progressive motion using the body first with the tail following in the direction of motion. Random changes in direction which occur without pause.

**Attached behaviours**	

A	Attached, but no motion

T	Slow tail movements from side to side or curling with body as point of attachment

T	Fast tail movements with body as point of attachment

h	Slow body movements with tail as point of attachment

H	Fast body movements with tail as point of attachment

b	Slow central body movements with the head of body and tail as a point of attachment

B	Fast central body movements with the head of body and tail as a point of attachment

### Molecular analyses

Cercarial pellets stored in ethanol were transferred onto Whatman FTA^® ^cards for DNA preparation carried out according to the manufacturer's protocol Whatman FTA^® ^cards. Polymerase chain reaction (PCR) amplifications were performed on a Geneamp PCR System 2700 (Applied Biosystems). Amplification of the mitochondrial cytochrome oxidase subunit 1 (CO1) was performed using 'ASMIT 1' (5'-3' TTTTTTGGGCATCTGAGGTTTAT) and 'ASMIT 2' (5'-3' TAAAGAAAGAACATAATGAAAATG) primers [24]. Amplifications were performed in 50 μl reactions containing one plug of Whatman FTA^® ^card extracted DNA, 1 μl of each primer (ASMIT 1 and ASMIT 2) at 50 pmolar concentration, 25 μl of HotStarTaq Master Mix (Qiagen) and 23 μl of dH_2_0. Thermal cycling was performed under the following conditions: 15 min at 95°C to activate the HotStarTaq DNA Polymerase and for denaturation, followed by 45 cycles of 15 sec at 94°C, 30 sec at 40°C and 45 sec at 72°C; followed by a final 7 min extension at 72°C. PCR products were purified in 96 well plates using a Millipore Montage PCR purification kit as per manufacturer's protocol. Sequencing was carried out using a BigDye Terminator v1.1 cycle sequencing kit (Applied Biosystems) in 10 μl reactions containing 3 ng of DNA for every 100 bps of PCR product, 1 pMols primer, 3 μl Big Dye dilution buffer (2.5×), 1 μl Big Dye reaction mix and xμl dH_2_0. Thermal cycling was performed under the following conditions: 5 min pre-denaturation at 96°C without Big Dye reaction mix, pause of cycler whilst samples transferred to ice and Big Dye added. Then 25 cycles of 10 secs were performed at 96°C, 5 secs at 50°C, and 4 mins at 60°C. Sequences were imported into Sequencher vs. 3.1.1. (GeneCodes corp.) and compared to control sample sequences in order to identify the species of schistosome cercariae present in each sample. Cercarial samples containing both species were identified by the presence of double peaks at nucleotide positions that differed between *S. mansoni *and *S. rodhaini *in the CO1 sequence.

### Statistical analyses

Differences in all parameters measured were tested for between lines using analysis of variance (in a generalised linear modelling procedure) in Minitab Release 14 (Minitab Inc., State College, PA). Experimental line was used as the independent variable. Dependent variables were the behavioural categories (see Table 1) and cercarial production for chronobiology. Dependent variables were transformed as necessary to meet the generalised model's assumptions of homogeneity of variance and normality of error. Where variables could not be sufficiently transformed, non-parametric Kruskal Wallace tests were used. As our effect sizes are clearly presented, and with appropriate metrics of their precision, we have chosen not to incorporate Bonferroni correction procedures here to control type-1 error rate; such procedures are increasingly viewed as unhelpful [25-28].

## Results

### Cercarial chronobiology (see figure 1)

**Figure 1:**
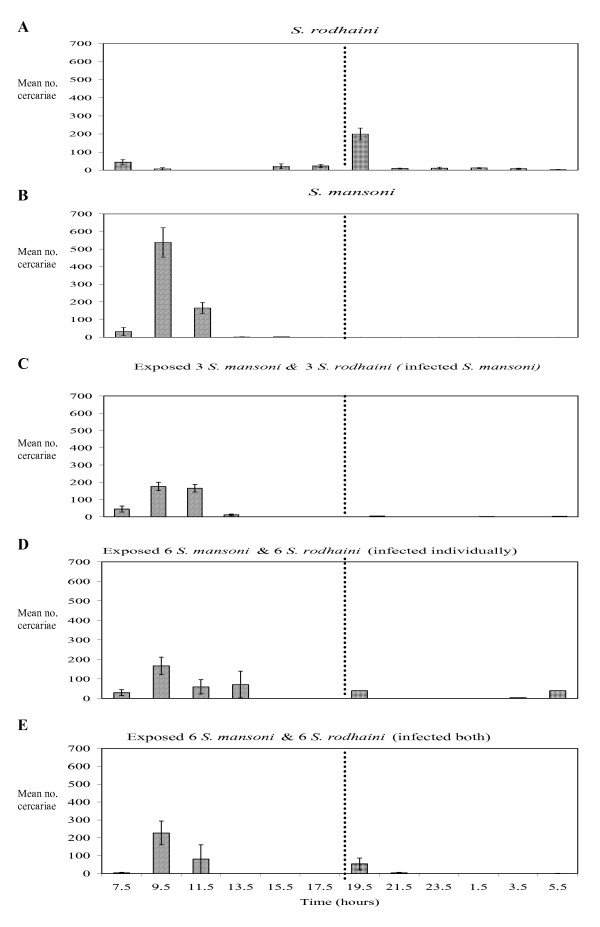
**Chronobiology of cercarial emergence**. Chronobiology of *S. mansoni *and *S. rodhaini *cercarial emergence from snails individually infected with each species (A and B), snails doubly exposed at a dose of 3 miracidia of each species but only patently infected with *S. mansoni *(C), doubly exposed at a dose of 6 miracidia of each species but with only single patent infections of *S. mansoni *or *S. rodhaini *(D) and snails doubly exposed to a dose of 6 miracidia of each species and doubly infected (E) over a 24 hr period (+- SE), ( . . . . = transition from light to dark.)

The *S. rodhaini *cercariae emerging from single species infections were shown to have an average nocturnal shedding peak of 200 ± 32 cercariae per snail between 1830–2030 hours. A low level of shedding continued throughout the night with an unpredicted small peak of 44 ± 14 cercariae per snail between 0630–0830 hours. *S. rodhaini*'*s *entire shedding period extended from 1430–1030 hours, however shedding was at a low level (<50 cercariae per snail) for all apart from the peak 2 hour period (1830–2030 hours). In contrast *S. mansoni *cercariae emerging from single species infections had an average diurnal shedding peak of 538 ± 84 cercariae per snail (significantly greater than that of *S. rodhaini *F_1,35 _= 16.64, p < 0.001) during the period 0830–1030 hours. The time period over which *S. mansoni *shedding occurred was very short 0630–1230 hours with negligible shedding (<5 cercariae per snail) for 4 hours thereafter.

For all snails exposed to both species of schistosome the species of cercaria shedding at each time point were successfully confirmed through genetic sequencing of their mitochondrial cytochrome oxidase subunit 1 (CO1). None of the snails exposed to 3 miracidia of *S. mansoni *and 3 miracidia of *S. rodhaini *simultaneously became patently infected with *S. rodhaini*. Those snails exposed to 6 miracidia of *S. mansoni *and 6 miracidia of *S. rodhaini *and confirmed to only be patently infected with *S. rodhaini *showed a similar emergence pattern to that of individual *S. rodhaini *infections, although the sample size was very small.

**Figure 2:**
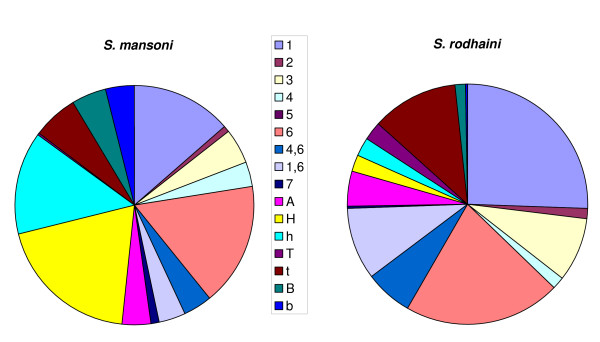
**Cercarial swimming behaviour**. Pie charts to show a comparison between the percentage of time unselected *S. mansoni *and *S. rodhaini *cercariae spend performing 16 different categories of behaviour (detailed in table 1).

Cercariae emerging from snails exposed to *S. mansoni *and *S. rodhaini *simultaneously at the two different doses, but only patently infected with *S. mansoni*, had lower cercarial production in addition to an apparent slight shift in diurnal rhythms than/from those from *S. mansoni *single species exposures. The peak of shedding that occurred between 0830–1030 hours for snails exposed to and patently infected with *S. mansoni *only was within the same time period for snails exposed to both parasites simultaneously but at a significantly lower level (176 ± 24 cercariae per snail for snails exposed to 3 miracidia of *S. mansoni *and 3 miracidia of *S. rodhaini *simultaneously (F_1,43 _= 25.78, p < 0.01) and 167 ± 45 cercariae per snail for snails exposed to 6 miracidia of *S. mansoni *and 6 of *S. rodhaini *simultaneously (F_1,22 _= 9.48, p = 0.006)) (Figure 2). A slight shift in the shedding pattern was observed with comparatively high levels of cercarial shedding occurring in the two hours after the peak (1030–1230 hours) (166 ± 21 cercariae per snail for snails exposed to 3 miracidia of *S. mansoni *and 3 miracidia of *S. rodhaini *simultaneously and 60 ± 36 cercariae per snail for snails exposed to 6 miracidia of *S. mansoni *and 6 of *S. rodhaini *simultaneously) in contrast to the large reduction in cercarial shedding for snails exposed to *S. mansoni *only in the two hours after the peak.

Three snails which were exposed to 6 miracidia of *S. mansoni *and 6 miracidia of *S. rodhaini *were identified to be patently infected with both species using molecular sequencing of the cercariae produced. The peak shedding periods for both species were at the same time (0830–1030 for *S. mansoni *and 1830–2030 for *S. rodhaini*) as for individual infections, however shedding was at lower level for both species from the mixed infection in comparison to single infections (227 ± 67 cercariae per snail for *S. mansoni *and 53 ± 33 cercariae per snail for *S. rodhaini*). The difference was significant for *S. rodhaini *(F_1,22 _= 5.03, p= 0.04) but not for *S. mansoni *(F_1,18 _= 2.38, p = 0.14). The time period over which shedding occurred was the same for *S. mansoni *cercariae emerging from mixed infections as for single infections (0630–1230 hours) and the pattern of emergence was similar. Molecular identification of cercariae revealed that the shedding period for *S. rodhaini *cercariae emerging from dually infected snails was restricted in comparison to snails only infected with *S. rodhaini *to 1830–2230 hours, with no shedding throughout the night or further peak in the early morning. This may be due to the significantly lower levels of shedding for *S. rodhaini *and resulted in no overlap in cercarial emergence of the two parasite species from co-infected snails.

### Cercarial swimming behaviour

#### Cercarial behavioural repertoire; a comparison between S. mansoni and S. rodhaini

Figure 2 gives a comparison of the average percentage of time cercariae from each species spent performing each individual behavioural category when produced from the individually exposed snail groups. No behavioural categories were unique to either species, although the proportion of time spent performing certain categories did differ significantly between the species. As these behavioural categories may not accurately represent those displayed under natural situations, due to the small amount of water in which they were videoed, further analyses were mainly performed on the groupings of behaviours into 'attached' or 'unattached' behavioural categories as defined in Table 1 in order to elucidate differences in the activity levels of the cercaria rather than individual behaviours. *S. mansoni *cercariae spent an approximately equal amount of time attached as unattached, significantly more time attached than *S. rodhaini *(H = 8.93, d.f.= 136, p = 0.003).

#### The effect of inter-specific competition on cercarial behaviour

A significantly greater amount of time was spent performing behaviour '7' (body first swimming) for both species with mixed species conditions (*S. mansoni *H = 6.65, d.f. = 160, p = 0.04; *S. rodhaini *H = 30.19, d.f. = 207, p < 0.001).

There was a significant increase in all attached behaviours for *S. rodhaini *under conditions of potential inter-specific competition (H = 22.99, d.f. = 136, p < 0.001 for 'H' (fast head movements), H = 24.65, d.f. = 136, p < 0.001 for h (slow body movements), H = 10.44, d.f. = 136, p = 0.005 for 't' (slow tail movements), H = 15.73, d.f. = 136, p < 0.001 for 'B' (fast body movements) and H = 19.43, d.f. = 136, p < 0.001 for 'b' (slow body movements)) except for 'T' (fast tail movements) where there was no significant difference. For *S. mansoni*, however, there were no significant changes in attached behaviour with species conditions.

## Discussion

This study has characterized both differences in, and effects of competition on, components of the cercarial phenotype related to host finding and infectivity for two closely related schistosome species. Molecular techniques have enabled, for the first time, subtle effects of inter-specific competition on chronobiology to be revealed and investigation of the cercarial attachment behaviour of both species has identified differences which may affect host finding behaviour.

In addition to confirming the diurnal shedding peak of *S. mansoni *and nocturnal shedding peak of *S. rodhaini *[17] an extra emergence peak of *S. rodhaini *was also identified in the early morning, consistent with that recently reported from the field in Kenya [16]. This peak could potentially lead to misidentification of *S. rodhaini *infections as *S. mansoni *in the absence of full 24 hour chronobiological investigation or molecular identification of parasites from field collected snails. Additionally, as this has recently been confirmed to be present in natural situations [16], it may lead to an increased likelihood of both parasite species co-infecting the same definitive hosts, allowing hybridization and mating competition between the two species to occur [9,13,15,16,21]. We also observed here that the *S. mansoni *shedding peak and indeed overall shedding was greater than that of *S. rodhaini *in this *B. glabrata *intermediate host, indicating that *S. mansoni *was more reproductively successful in this host than *S. rodhaini*.

Inter-specific competition was shown to influence cercarial chronobiology with co-infection resulting in a slight shift in the *S. mansoni *shedding pattern and a reduction of the *S. rodhaini *shedding period (potentially as a result of the observed general reduction in the number of cercaria shed), in comparison to single species infections, preventing overlap and therefore possible interference with *S. mansoni *shedding. Importantly this effect on chronobiology does not result in a large shift for either species and is unlikely therefore to affect the species of hosts available for infection by cercariae on emergence. This effect competition would not have been identified without the use of molecular characterisation of emerging cercariae as it occurred in a period of overlap in shedding of the two species. It demonstrates that although previous studies have shown chronobiology to be genetically determined [17,18] there is also some phenotypic plasticity.

In addition to influencing the time at which cercariae were released, the chronobiological studies revealed that both co-exposure with and without patent co-infection resulted in lower hourly cercarial production for both parasite species in comparison to single species exposures and infections. This reduction was seen for cercariae produced from those snails exposed to three miracidia as well as six of each parasite species simultaneously, therefore excluding miracidial dose as an explanation for the difference in cercarial production and implicating inter-specific competition. Indeed parasite species co-infecting the same host are predicted to undergo competition for shared resources [29] and in this interaction it appears that such competition has negative effects on the asexual reproduction of both the interacting species.

Cercarial activity is known to relate to infectivity [7,8]. The main difference observed between swimming behaviours for *S. mansoni *and *S. rodhaini *was that *S. mansoni *cercariae spent a significantly greater amount of time attached to the petri dish than *S. rodhaini *cercariae. The causality/functionality of such attachment behaviour is unclear, and of course the possibility of experimental artefact cannot be fully discounted here. However, whether an artefact of this particular laboratory set-up or not, in a previous study time spent attached was shown to be a very useful variable negatively correlated to infectivity to the definitive host [7]. Additionally, the behavioural category identified in this study of 'body first swimming', which *S. mansoni *performed for a significantly greater amount of time than *S. rodhaini*, has been identified in previous studies where it has been associated with active attachment of a cercaria to a substrate [30]. The lack of post attachment searching behaviour may indicate that such active attachment was performed to allow the cercariae to rest [31], rather than try to penetrate the substrate. The difference in time spent attached between *S. mansoni *and *S. rodhaini *is therefore likely to represent different strategies of energy conservation between the two species, with *S. mansoni *conserving its energy in the absence of host cues [5,6]. No exclusive behavioural categories were identified for either species in this study to allow differentiation between the species through their swimming behaviour. Further investigation in order to directly link phenotypic behaviours of cercariae in natural conditions to the parasite's definitive host species is warranted.

Inter-specific competition was shown to influence these parasite swimming behaviours. The reduction in active swimming behaviours and increase in body first swimming exhibited by both species and increase in attachment time for *S. rodhaini *exhibited under conditions of potential inter-specific competition may reflect reductions in cercarial glycogen levels due to the detrimental effects of inter-specific competition. The lack of difference in amount of time spent attached by *S. mansoni *cercariae emerging from co-exposed snails in comparison to individually exposed snails may indicate that competition between the two parasite species had less of a detrimental effect on *S. mansoni *than *S. rodhaini*.

## Conclusion

In conclusion, through investigation of the phenotype of such a readily available and abundant life-stage of the schistosome life-cycle, we have gained important insights into the complexities of this medically important host-parasite system. Inter-specific competition between these schistosome species has been implicated in influencing cercarial production, chronobiology and activity. In natural geographic areas where these two species interact they can be identified with some certainty by comprehensive investigation of their chronobiological shedding peaks, which remain distinct under competition. Further investigation of the effects of inter-specific competition on these phenotypes in the field is now required.

## Competing interests

The authors declare that they have no competing interests.

## Authors' contributions

AJN conducted all the experiments and analyses and wrote the manuscript. DR helped to design the experiments, participated in its coordination and provided substantial revisions to the manuscript. LR analysed the cercarial videos and helped with the writing of the manuscript. JPW led the design of the experiments, provided close supervision throughout and substantial contribution to the manuscript.
